# A Facile Two-Step Hydrothermal Synthesis of Co(OH)_2_@NiCo_2_O_4_ Nanosheet Nanocomposites for Supercapacitor Electrodes

**DOI:** 10.3390/nano13131981

**Published:** 2023-06-30

**Authors:** Hammad Mueen Arbi, L. Vijayalakshmi, Yedluri Anil Kumar, Salem Alzahmi, Chandu V. V. Muralee Gopi, Andrivo Rusydi, Ihab M. Obaidat

**Affiliations:** 1 Department of Physics, United Arab Emirates University, Al Ain P.O. Box 15551, United Arab Emirates; 201990023@uaeu.ac.ae; 2 Department of Automotive Engineering, Yeungnam University, Gyeongsan-si 38541, Republic of Korea; lvl.phy@gmail.com; 3 Department of Chemical & Petroleum Engineering, United Arab Emirates University, Al Ain P.O. Box 15551, United Arab Emirates; yedluri.anil@gmail.com; 4 National Water and Energy Center, United Arab Emirates University, Al Ain P.O. Box 15551, United Arab Emirates; 5 Department of Electrical Engineering, University of Sharjah, Sharjah P.O. Box 27272, United Arab Emirates; naga5673@gmail.com; 6 Advanced Research Initiative for Correlated-Electron Systems (ARiCES), Department of Physics, National University of Singapore, Singapore 117551, Singapore; phyandri@nus.edu.sg

**Keywords:** Co(OH)_2_@NiCo_2_O_4_, hydrothermal route, supercapacitors, electrochemical performance, energy storage

## Abstract

The composites of NiCo_2_O_4_ with unique structures were substantially investigated as promising electrodes. In this study, the unique structured nanosheets anchored on nickel foam (Ni foam) were prepared under the hydrothermal technique of NiCo_2_O_4_ and subsequent preparation of Co(OH)_2_. The Co(OH)_2_@NiCo_2_O_4_ nanosheet composite has demonstrated higher specific capacitances owing to its excellent specific surface region, enhanced rate properties, and outstanding electrical conductivities. Moreover, the electrochemical properties were analyzed in a three-electrode configuration to study the sample material. The as-designed Co(OH)_2_@NiCo_2_O_4_ nanosheet achieves higher specific capacitances of 1308 F·g^−1^ at 0.5 A·g^−1^ and notable long cycles with 92.83% capacity retention over 6000 cycles. The Co(OH)_2_@NiCo_2_O_4_ nanosheet electrode exhibits a long life span and high capacitances compared with the NiCo_2_O_4_ and Co(OH)_2_ electrodes, respectively. These outstanding electrochemical properties are mainly because of their porous construction and the synergistic effects between NiCo_2_O_4_ and Co(OH)_2_. Such unique Co(OH)_2_@NiCo_2_O_4_ nanosheets not only display promising applications in renewable storage but also reiterate to scientists of the unlimited potential of high-performance materials.

## 1. Introduction

Unprecedented fossil fuel consumption became one of the most significant global challenges due to the ongoing increase in the world’s population [1,2,3]. Facing this critical issue of fossil fuel depletion as well as the climate emergency crises, the petition for synthesizing eco-friendly and renewable energies became more and more important [4,5,6]. Hence, new energy storage and harvesting technologies became the focus of ongoing global research. Supercapacitors (SCs) and batteries were among the energy-storing devices that significantly improved for better energy-harvesting devices [7,8]. Batteries are typically energy-storing assets that use faradaic electrochemical procedures to store electrical energy and release it on demand, albeit with a limited cycling span [9,10]. The attempts toward improving the cycling span of the batteries have led to the pioneering of SCs [11]. SCs are mainly classified as electrochemical double-layer capacitors (EDLCs) and pseudocapacitors (PCs) [12]. The sample electrodes utilized for EDLCs are carbon-type elements that include graphene, carbon nanotubes (CNTs), graphite, etc. These electrodes possess an enlarged specific surface [13,14,15].

Transition metal oxides (TMOs), a representative class of PC nanomaterials, have captured a lot of attention due to their abundant complexity valence state and their rapidly reversible procedures. Among them, RuO_2_ material possesses a standard TMO status and achieves excellent energy storage properties, but it has serious limitations due to its notably high prices and large toxicities. [16]. Surprisingly, spinel NiCo_2_O_4_ is a promising candidate for conventional TMOs, offering superior benefits in electrode samples due to its cost-effective and efficient electrochemical process [17,18]. One out of three Co atoms in Co_3_O_4_ is replaced with a Ni atom [19,20,21]. The NiCo_2_O_4_ structure is ordered as there are two metal sites in this structure, one being tetrahedral (the 8a site) and the other being octahedral (the 16d site) [22,23,24]. Moreover, the Ni metallic ions would produce plentiful redox reactions to achieve energy storage activities in contrast to Co_3_O_4_ [25,26].

NiCo_2_O_4_ was rarely noticed as the energy material for the electrode composites of SCs due to its restricted capacities (407 F·g^−1^) [27]. On the other hand, Co(OH)_2_ was one of the classic PC electrodes, displaying superior theoretical capacities (3460 F·g^−1^), and well-redox reactions, in addition to being a hydrotalcite-type compound [28]. The expanded interlayer spaces could supply superior specific surfaces to transport the ions/electrons rapidly, which, in turn, enhances the electrochemical activities [29]. However, Co(OH)_2_ displays lower specific capacity and low-level cycling stabilities behavior [30,31]. These limits were ascribed to the relatively very low conductivities and correspondingly fewer availabilities of active sites [32,33,34]. To achieve better performances, Co(OH)_2_ has been designed into a microscale composite with a conductive foam. In this regard, the as-developed electrode materials consist of expanded specific surface areas, shorter electron transport paths, and plenty of energy-storing active sites. By combining NiCo_2_O_4_ and Co(OH)_2_, the capacitance of NiCo_2_O_4_ can be improved. Evidently, a combination of Co(OH)_2_@NiCo_2_O_4_ with a conductive matrix-type nickel foam can enhance the cycling performances and rate capabilities of materials. To the best of our understanding, the capabilities of a Co(OH)_2_@NiCo_2_O_4_ were not adequate, and it was the main endeavor to further boost the electrochemical activities of SCs. Compared to the recently published composites, it would be anticipated that the Co(OH)_2_@NiCo_2_O_4_ might produce stronger cooperatives effects, encouraging enhanced electrochemical performances for SCs.

By combining the excellent electrochemical performances of Co(OH)_2_ and the high conductivity of NiCo_2_O_4_, a unique nano-structure of Co(OH)_2_@NiCo_2_O_4_ was in situ assembled on Ni foam as a binder-free sample. This composite material not only forms an open inter-network for easier ions diffusion but also enhances the specific area to offer abundant electro-active sites for the Faradic procedures. NiCo_2_O_4_ nanoparticles as core nanomaterial were developed using a hydrothermal procedure where the Co(OH)_2_ shell could be simply coated onto the interface of NiCo_2_O_4_ nanoparticles via a hydrothermal route with high efficacy. Co(OH)_2_ nanomaterial, when made as a nanocomposite with NiCo_2_O_4_, offers improved electrochemical reactions due to its expanded surface region, hierarchical morphologies, well mechanical properties, good chemical stabilities, and excellent electrical kinetics. The as-designed Co(OH)_2_@NiCo_2_O_4_ nanosheet achieved high specific capacitances of 1308 F·g^−1^ at 0.5 A·g^−1^ with 92.83% capacity retention over 6000 cycles. Thus, we deduce that combining Co(OH)_2_ with NiCo_2_O_4_ effectively improved the kinetics of the composite material, the specific capacitances, the cycling stabilities, and the rate capabilities of SCs.

## 2. Experimental

### 2.1. Synthesis of Co(OH)_2_@NiCo_2_O_4_ Nanosheet Composite

The chemicals used in this study were purchased from Sigma-Aldrich, Busan, Republic of Korea, and utilized without additional purification; these are potassium hydroxide (KOH), cobalt nitrate hexahydrate (Co(NO_3_)_2_·6H_2_O), ammonium hydroxide (NH_4_OH), nickel nitrate hexahydrate (Ni(NO_3_)_2_·6H_2_O), hydrochloric acid (HCl), and urea (CH_4_N_2_O). A clean Ni foam (with a size of 2 × 4 cm^2^) was immersed into a mixed solution of 10 mmol Co(NO_3_)_2_, 20 mL of 28 wt% ammonia solution, 15 mmol NH_4_F, and 25 mL of H_2_O. The upside of the Ni foam was covered from solution contaminations by uniformly coating it with polytetrafluorethylene tapes. The autoclave was collected and put at 130 °C for 12 h for nanoparticle growth. Then, the product was collected and rinsed multiple times with DI water to detach the residual particle debris. 

**Scheme 1:**
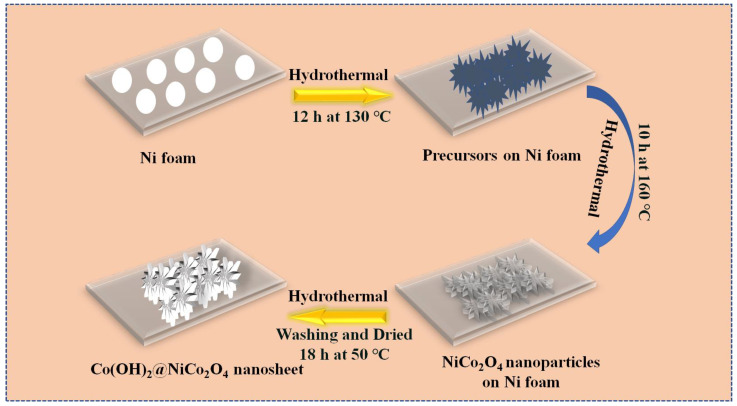
Schematic illustration of the synthesis of Co(OH)_2_@NiCo_2_O_4_ nanosheet composite electrode.

The Co(OH)_2_@NiCo_2_O_4_ electrodes were fabricated via an easy one-step hydrothermal route. All the reagents were analytically graded and organized without further purifications. In the particular fabrication experimental part, 0.94 g of Co(NO_3_)_2_·6H_2_O and 0.503 g of Ni (NO_3_)_2_.6H_2_O were dissolved in 30 mL DI water using a stirrer. The relevant quantity of polyethylene glycol was dissolved in 15 mL DI water using a stirrer. The precursors of Ni^2+^ and the Co^2+^ solutions were included in the solution of polyethylene glycol concurrently under a continuous magnetic stirrer. Then, 25 mL ammonium hydroxide was included in the above mixture solutions. After continuous stirring for 45 min to form a final mixture solution, the final suspension precursor was shifted into a 70 mL autoclave and optimized at 160 °C for 10 h. Then, after the autoclave came to normal temperature, the final products were gathered, washed, and dried at 50 °C for 18 h. Scheme 1 described the information on the synthesis of Co(OH)_2_@NiCo_2_O_4_ nanosheet composited electrode. 

### 2.2. Physical Characterization

The crystallinity and structure of Co(OH)_2_@NiCo_2_O_4_ nanosheet were studied via an XRD analysis (Rigaku, XRD, Bruker D8 Advance, a wavelength of 1.540 Å, Bruker AXS LTD., Busan, Republic of Korea). The microstructures of the developed nanomaterials were studied using FE-SEM (FE-SEM, JSM-7800F equipping, JEOL LTD., Busan, Republic of Korea) instrument operated at an accelerating voltage of 5 kV. The composite elements were studied using energy-dispersive X-ray (EDAX, BRUKER, Thermo Scientific LTD., Busan, Republic of Korea) equipment. A high-resolution transmission electron microscope (TEM, JEM-2100F, JEOL LTD., Busan, Republic of Korea) instrument operated at an accelerating voltage of 200 kV was employed to find the HR-TEM images of the grown Co(OH)_2_, NiCo_2_O_4_ and Co(OH)_2_@NiCo_2_O_4_ nanosheet. The valence states of the developed nano-constructions were registered using an X-ray photoelectron spectrometer (XPS, ESCCALAB 250Xi, Thermo Scientific LTD., Busan, Republic of Korea) with a maintaining power of 60 W.

### 2.3. Electrochemical Characterization

The electrochemical properties were achieved using a 3-electrode configuration with Co(OH)_2_@NiCo_2_O_4_ nanosheet, Ag/AgCl, and platinum as the working, reference, and counter electrodes, respectively, in an electrolyte of 2 M KOH solution using the electrochemical (Bio-Logic Model: SP-150, Busan, Republic of Korea) equipment. The galvanostatic charge/discharges (GCD) analyzer was conveyed in the window of 0.0 to 0.5 V at a current density in the range of 10–50 A·g^−1^. The cyclic voltammetry (CV) examinations were carried out in the window ranges from 0.0 to 0.5 V at scan rates in the range of 0.5–10 mV·s^−1^. The electrochemical impedance spectroscopy (EIS) measurements were performed at several frequencies from 1 mHz to 100 kHz at a magnitude of 15 mV. By using GCD plots, the specific capacitances were determined by the following formula [35,36]:

(1) $$C_{sc}=\frac{I\times \Delta t}{m\times \Delta v}$$

where *I* is the current (A), ∆*t* is the discharging time (secs), *m* is the mass of sample (g), and ∆*V* is the potential (V).

## 3. Results and Discussions

**Figure 1:**
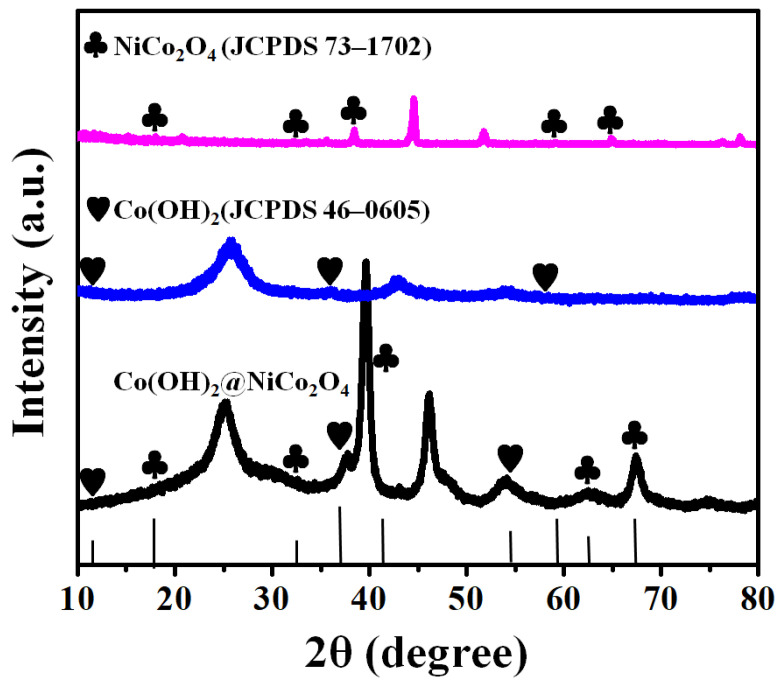
XRD pattern of as-synthesized Co(OH)_2_@NiCo_2_O_4_ nanosheet grown on Ni foam.

Figure 1 demonstrates the XRD spectra of the as-grown samples. For all the electrodes, the main diffraction points at 2θ values of 44.5°, 51.9°, and 76.5° are due to the Ni skeleton. Diffraction peaks at 2θ = 18.79°, 35.39°, 36.87°, 62.35°, and 64.83° correspond to (220), (311), (222), (440), and (531) facets, respectively. They were effectively indexed with the spinel spacing of NiCo_2_O_4_ (JCPDS card no. 73–1702) in good correlation with recent reports [37]. Diffraction peaks located at 2θ = 11.2°, 33.1°, and 59.73° correspond to (003), (012), and (110) facets, which were well-marked with the Co(OH)_2_ phases (JCPDS card no. 46-0605) [38,39]. It is noticed that there are very slight peaks shifts of NiCo_2_O_4_ and Co(OH)_2_ phases. We believe that these peak shifts could be due to the existence of some stress at the Co(OH)_2_@NiCo_2_O_4_ interface. Thus, the as-prepared sample was confirmed to be a composite of NiCo_2_O_4_ and Co(OH)_2_. All angle diffraction facets provided evidence for the admirable crystallinity of Co(OH)_2_@NiCo_2_O_4_ nanosheet composite on Ni foam. 

FTIR spectra of the as-obtained NiCo_2_O_4_ electrode and Co(OH)_2_@NiCo_2_O_4_ nanosheet are recorded and shown in Figure S2. It can be seen that the characteristic peaks of NiCo_2_O_4_ appear at about 553 and 643 cm^−1^ (Figure S2a), belonging to metal–oxygen M−O vibrations of the NiCo_2_O_4_. The peaks at around 3413 and 1628 cm^−1^ are attributed to the vibrational mode of absorbed H_2_O, and the peak at 1383 cm^−1^ may be associated with the presence of physically adsorbed CO_2_. In contrast, in the spectrum of Co(OH)_2_@NiCo_2_O_4_ nanosheet (Figure S2b), the characteristic peaks of NiCo_2_O_4_ appear at about 560 and 648 cm^−1^, showing slightly red-shift compared with the pure NiCo_2_O_4_. Our observation of both the difference in peak position and intensity between the as-prepared Co(OH)_2_@NiCo_2_O_4_ nanosheet and the pure NiCo_2_O_4_ reported in the literature indicates a mutual interaction between Co(OH)_2_ and NiCo_2_O_4_, further confirming that the Co(OH)_2_ has been coated closely on the NiCo_2_O_4_ electrode surface. 

**Figure 2:**
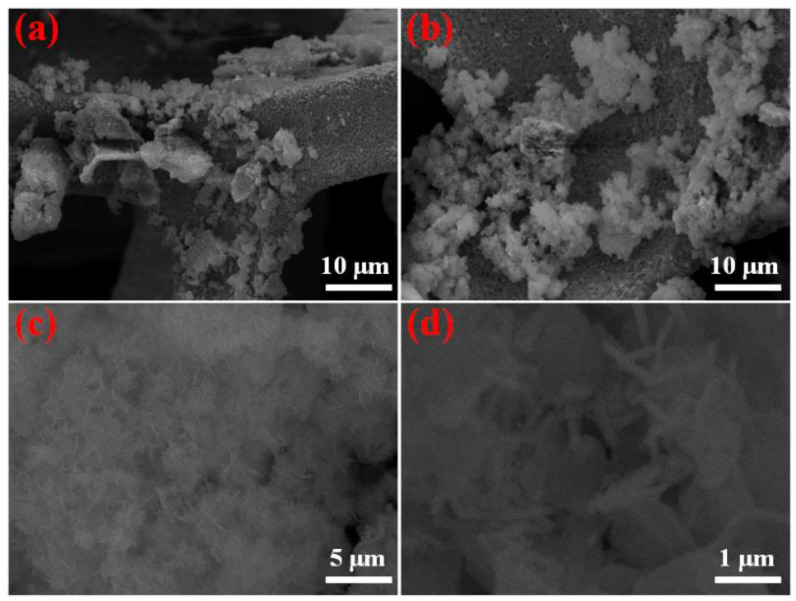
SEM images of (**a**) NiCo_2_O_4_ nanoparticles, (**b**) low-magnification of Co(OH)_2_@NiCo_2_O_4_ nanosheet, and (**c**,**d**) high-magnification images of Co(OH)_2_@NiCo_2_O_4_ nanosheet composite grown on Ni foam.

The SEM mappings (Figure 2) of the electrode material demonstrate the morphologies of the samples. Figure 2a discloses the nanostructure of pure NiCo_2_O_4_ nanoparticles that consist of major tips and a length of about 10 μm. The nanoparticles were collected from many smaller particles to make porous constructions. The mesoporous in NiCo_2_O_4_ nanoparticles would enable the ion diffusions, which further enhances the electrochemical activities. Figure 2b displays the low-magnification SEM image of the Co(OH)_2_@NiCo_2_O_4_ nanosheet composite. When compared with binary NiCo_2_O_4_ nanoparticles, the surface shifts from smooth to rough with the coating of Co(OH)_2_ nanoparticles (Figure 2c). The coatings were mesoporous structures that were networked with each other to form an excellent facilitator surface area. In particular, the Co(OH)_2_ particles were significantly anchored onto NiCo_2_O_4_ nanoparticles as backbones to construct a unique core–shell morphology (Figure 2d). The porous NiCo_2_O_4_ nanoparticles and Co(OH)_2_ particles make the interior of the composite nanosheets feasible for the electrolytes. From Figure 2, the elemental mapping images of Co, Ni, and O elements once again confirm the formation of Co(OH)_2_@NiCo_2_O_4_. Atomic percentages of Co, Ni, and O are obtained to be 19.41%, 43.56%, and 29.12%, respectively. Their atomic molar ratio is thus about 2:5:2. The detailed explanation is given in the revised manuscript. These are percentages normalized to 100% as the sum of percentages for elements detected and quantified. Not all elements, such as very light ones, are detected and therefore are not included in the 100%. Also, elements present in a small quantity, even, e.g., carbon, etc., below about 0.3 wt% cannot be quantified or are quantified with big uncertainty.

**Figure 3:**
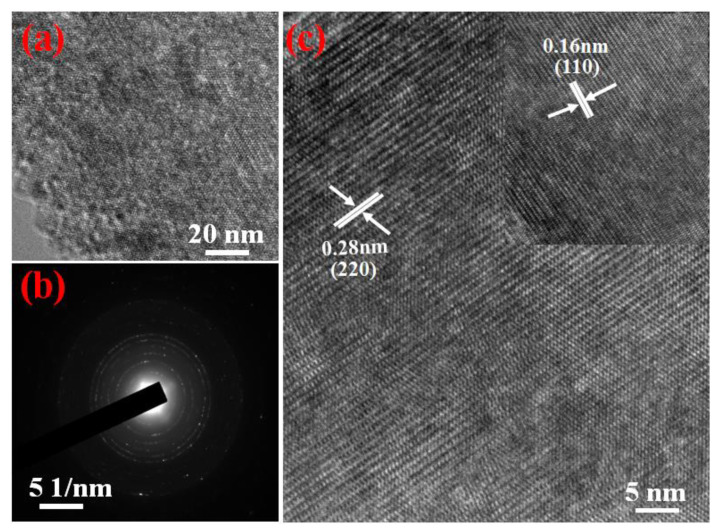
(**a**) TEM, (**b**) SAED pattern, and (**c**) HRTEM images of the composite Co(OH)_2_@NiCo_2_O_4_ nanosheet electrode grown on Ni foam.

TEM measurements (Figure 3) were carried out to disclose the comprehensive structure of the as-designed samples. Figure 3a illustrates the TEM images, (b) the SAED pattern, and (c) the HRTEM image of the Co(OH)_2_@NiCo_2_O_4_ nanosheet composite. Figure 3a shows the NiCo_2_O_4_ particle formations, indicating that the single crystalline nature of the material is porous and constructed with many irregular particles, which correlated with SEM data. Meanwhile, the pattern of SAED reveals the polycrystalline (co-centric rings) nature of the samples of NiCo_2_O_4_ [34]. The good-indexed lattice fringe with interplanar spaces of 0.28 nm was ascribed to the (220) facets of the NiCo_2_O_4_ spinel angle. This demonstrates further evidence of the formation of crystallized nickel cobaltite. 

**Figure 4:**
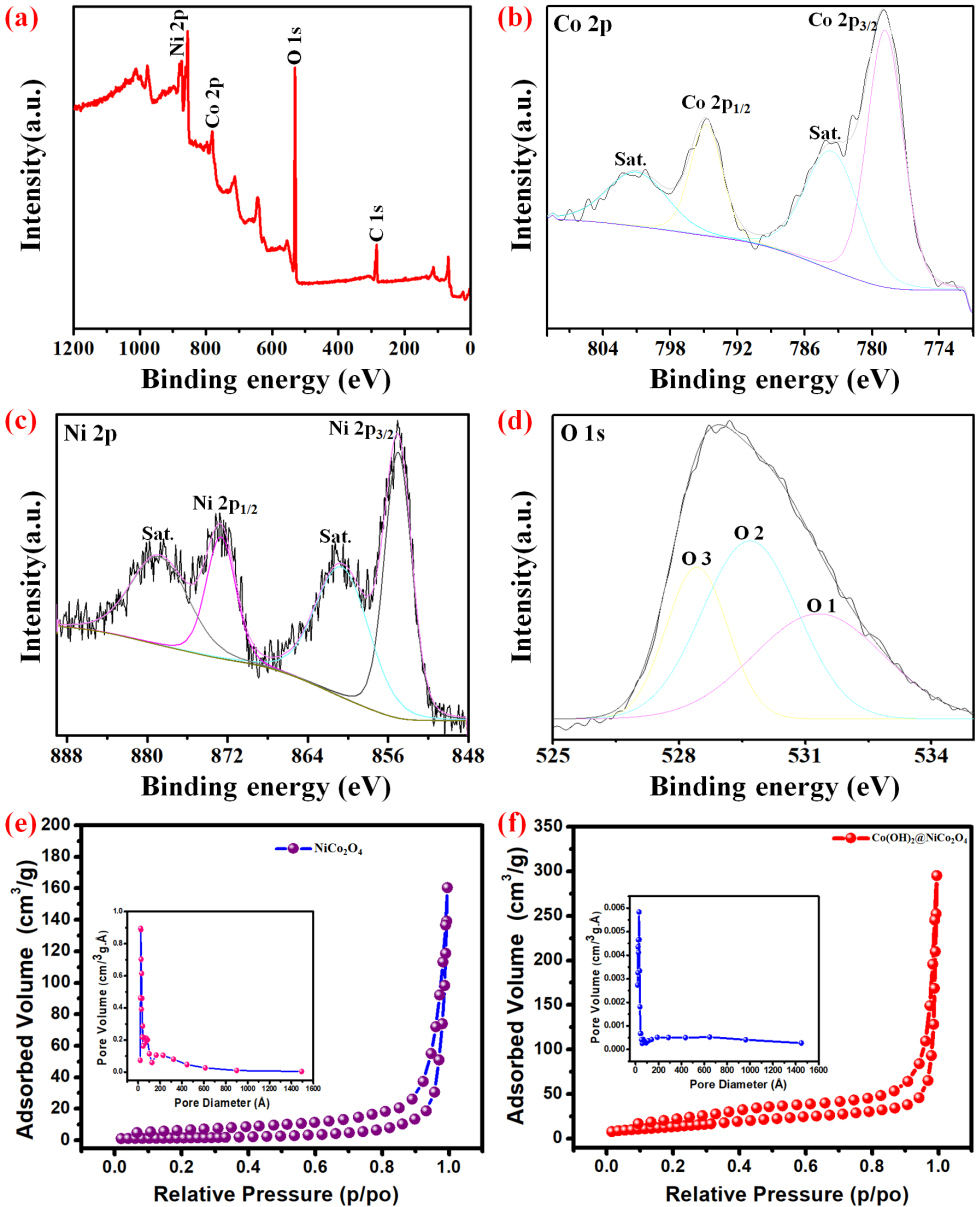
XPS spectra Co(OH)_2_@NiCo_2_O_4_ nanosheet composite of (**a**) full survey spectrum, (**b**) Ni 2p, (**c**) Co 2p, and (**d**) O 1s, respectively, and (**e**,**f**) N_2_ adsorption/desorption isotherms and pore diameter distributions of NiCo_2_O_4_, Co(OH)_2_@NiCo_2_O_4_.

XPS analysis was performed to investigate the oxidational elements and valence states of the as-designed samples, which are displayed in Figure 4. The full spectra (Figure 4a) reveal only the existences of Ni, Co, C, and O elements, which correlates with the XRD results. The major peaks from Co and the weaker peaks from Ni are due to NiCo_2_O_4_ being entirely covered by Co(OH)_2_ particles. Figure 4b illustrates two strong peaks originating at 797.4 eV and 780.7 eV, which are attributed to Co 2p_1/2_ and Co 2p_3/2_, respectively. The other satellite points are noticed at 803.1 eV and 786.4 eV. The fitting of Co 2p peaks at 780.6 eV and 796.4 eV are indexed to Co^3+^, and the fitting of Co 2p peaks at 782.5 eV and 797.7 eV correspond to Co^2+^. The data also were correlated to the Co spectrum of Co(OH)_2_ in recent notices [40]. In Figure 4c for Ni 2p spectra, excluding two satellite points, the peaks pointed at 873.6 eV and 855.7 eV for Ni 2p_1/2_ and Ni 2p_3/2_, respectively, were assigned to Ni^2+^, while the other two peaks at 875.7 eV and 856.8 eV correlate with Ni^3+^ [41,42]. The O 1s spectrum was deconvoluted (Figure 4d) into three peaks, namely O1, O2, and O3. The O1 at 530.1 eV binding energy supports the oxygen–metals bonding, the O2 at 531.4 eV is ascribed to oxygen in the OH^−^ group and defect sites, whereas O3 at 531.4 eV represents the –H–O–H content absorbed at the sample [43,44]. As displayed in Figure 4d, the XPS spectrum was separated into three peaks around 527.7 eV, 529 eV, and 530 eV, which were assigned to Al_2_O_3_ (36%), hydroxides (47%), and carbonates (17%) from the side of lower binding energy, respectively. Through the XPS and XRD outcomes, we can confirm that the as-prepared sample was the Co(OH)_2_@NiCo_2_O_4_ composite. Figure S1 displays the XPS full survey spectrums of Co(OH)2 and NiCo2O4 electrode materials. The full spectra (Figure S1a) reveal only the existence of Co and O elements in the Co(OH)_2_ material. The full spectra (Figure S1b) reveal only the existence of Ni, Co, and O elements in the NiCo_2_O_4_ electrode material. 

Figure 4e shows that the isotherms of NiCo_2_O_4_ display a type-IV mesopore feature with a hysteresis loop in the middle-pressure area. Their pore size distribution values are consistent with the isotherms. On the other hand, Figure 4f shows the isotherm of Co(OH)_2_@NiCo_2_O_4_ nanosheet composite, which was a typical type-I isotherm owing to the sharp increase at a lower relative pressure (P/P_0_ < 0.1), which reveals the existence of micropores. The isotherm adsorption line illustrates a vital hysteresis loop in the middle-pressure part (P/P_0_ = 0.4–0.9), which was due to the condensation of N_2_ molecules under lower pressure sections, and then the pores are occupied. The pore size distribution of Co(OH)_2_@NiCo_2_O_4_ nanosheet composite describes two peaks at 1.4 nm and 4.2 nm, which further confirms the presence of micropores and mesopores. The BET surface areas are measured to be 54.6 m^2^·g^−1^, and 41.3 m^2^·g^−1^ for Co(OH)_2_@NiCo_2_O_4_, and NiCo_2_O_4_, respectively. The Co(OH)_2_@NiCo_2_O_4_ nanosheet composite had the highest surface area of the three samples and is thus expected to have excellent electrochemical performance. 

### Electrochemical Properties

**Figure 5:**
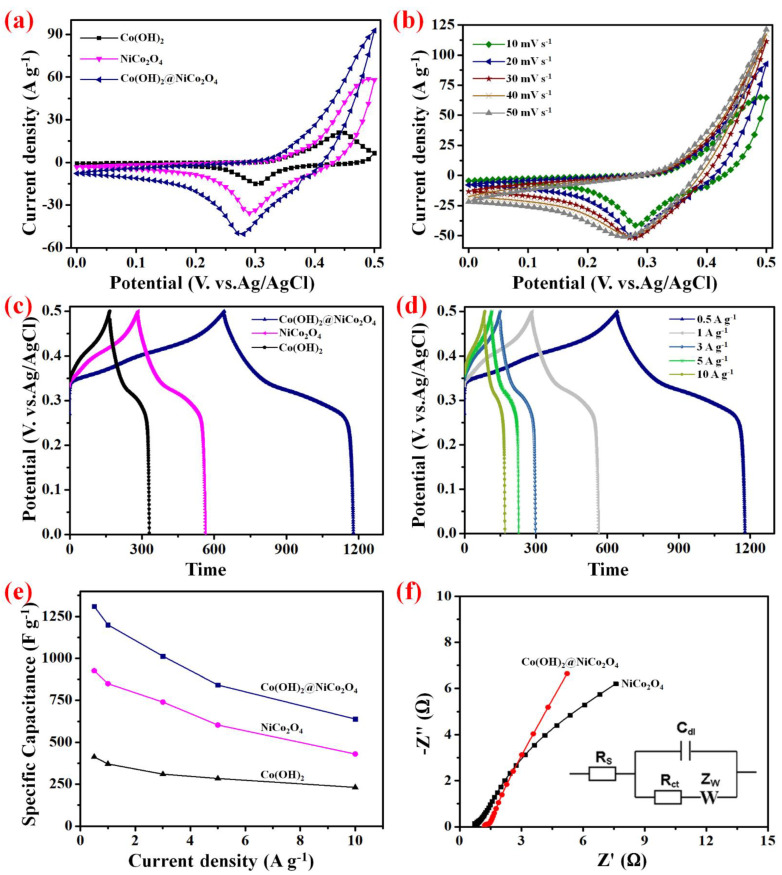
(**a**) CV curves of electrodes at 10 mV·s^−1^, (**b**) CV curves of composite Co(OH)_2_@NiCo_2_O_4_ nanosheets electrode at various scan rates, (**c**) GCD curves of electrodes at 0.5 A·g^−1^, (**d**) GCD curves of composite Co(OH)_2_@NiCo_2_O_4_ nanosheets electrode at different current densities, (**e**) specific capacitance of electrodes at various current densities, and (**f**) Nyquist plots for the electrodes.

The electrochemical properties of the Co(OH)_2_, NiCo_2_O_4_, and Co(OH)_2_@NiCo_2_O_4_ nanosheet electrode samples were analyzed using a three-electrode cell in an electrolyte of 2 M KOH solution at the ambient temperature. The CV plots were maintained within the potential window of 0.0–0.5 V. Figure 5a demonstrates the CV measurements of various samples at fixed scan rates, e.g., at 5 mV·s^−1^. The noticeable faradic peaks reveal redox reactions. The faradic procedures are given by the following formulas [45]:

Co(OH)_2_ + OH^−^ ⇔ CoOOH + H_2_O + e^−^ (2)

NiCo_2_O_4_ + OH^−^ + H_2_O ⇔ NiOOH + 2CoOOH + e^−^ (3)

CoOOH + OH^−^ ⇔ CoO_2_ + H_2_O + e^−^ (4)

A pairing of redox peaks appeared in the plot of the NiCo_2_O_4_ sample, which is due to the faradic reactions of Co^4+^/Co^3+^ and Ni^3+^/Ni^2+^. In the Co(OH)_2_ sample, two couples of faradic peaks Co^4+^/Co^3+^ and Co^3+^/Co^2+^ were revealed in Equations (2)–(4). Thus, the Co(OH)_2_@NiCo_2_O_4_ nanosheet composite electrode discloses coupling pairs of redox peaks. Figure 5b demonstrates the CV plots of the Co(OH)_2_@NiCo_2_O_4_ nanosheet composite at various scan rates. The reduction and oxidation points switch significantly toward positive and negative windows with increased scans rate because the diffusion outlay of the electrode ions decreases at high scan rates, which prevents ions adsorptions/desorption.

Figure 5c illustrates the GCD plots of the same samples under a current density rate in the range of 0.5–10 A·g^−1^, while the voltage window ranges from 0.0 to 0.5 V to acquire direct comparisons. As anticipated, the composite Co(OH)_2_@NiCo_2_O_4_ nanosheet electrode consists of prolonged discharge times (573 s) compared with the remaining electrodes of NiCo_2_O_4_ (303 s) and Co(OH)_2_ (196 s). At 0.5 A·g^−1^, according to Equation (1), the composite Co(OH)_2_@NiCo_2_O_4_ nanosheets electrode possesses the higher specific capacitances of 1308 F·g^−1^, while for NiCo_2_O_4_, it was 926 F·g^−1^, and for Co(OH)_2_, it was 413 F·g^−1^. 

These outcomes reveal that the composite Co(OH)_2_@NiCo_2_O_4_ nanosheet electrode possesses greater specific capacitances than the other Co(OH)_2_ and NiCo_2_O_4_ electrodes. Moreover, the growth material of Co(OH)_2_ not only supplies plentiful electroactive sites but also possesses open networks for electrolytes to facile transportations. In addition, the high conductivity of NiCo_2_O_4_ resulted in a reduced resistance between the Co(OH)_2_ electrode and Ni foam, improving the integral conduciveness of the composite sample. The uniform growth Co(OH)_2_ on NiCo_2_O_4_ nanoparticles efficiently resulted in the aggregations of Co(OH)_2_ particles. 

The GCD plots of the as-developed composite electrode at different current densities from 0.5 to 10 A·g^−1^ are depicted in Figure 5d. Figure 5e outlined the dependences of capacitances on current densities, indicating the rate activities of the sample materials. The specific capacitances of composite Co(OH)_2_@NiCo_2_O_4_ nanosheets electrode were recorded to be 1308, 1101, 1013, 842, and 639 F·g^−1^ at 0.5, 1, 3, 5, and 10 A·g^−1^, respectively, which are much greater than those of Co(OH)_2_ and NiCo_2_O_4_ electrodes. The capacitances of the electrode materials significantly decrease as the current increases because some electrons will not be able to completely access the interior of the samples. Figure S3a,b displays CV and GCD plots of pure NiCo_2_O_4_ electrodes at various scan rates and different current densities grown on Ni foam. 

To investigate the kinetic reaction of the electrode samples, EIS analysis was performed at a frequency from 100 kHz to 0.01 Hz. Figure 5f displays the Nyquist plots of all samples. Each curve consists of an inclining line in the lower frequencies area and a mini semicircle in the higher-frequencies zone [46,47,48]. As per the equivalent circuit diagram, which consists of equivalent series resistance (Rs), charge-transfer resistance (Rct), and Warburg resistances (W), the composite Co(OH)_2_@NiCo_2_O_4_ nanosheet sample exhibits a small outcome (0.913 Ω) compared with the NiCo_2_O_4_ (1.763 Ω) and Co(OH)_2_ (2.137 Ω) electrodes. For the composite Co(OH)_2_@NiCo_2_O_4_ nanosheets materials, Rct obtained from the partial semicircle is 0.096 Ω, while the Rct values for NiCo_2_O_4_ and Co(OH)_2_ samples are 0.172 Ω and 0.181 Ω, respectively. These values signify that composite Co(OH)_2_@NiCo_2_O_4_ nanosheet electrodes possess greater conductivities. The inclination of the plots in lower frequencies indicates the diffusion resistivity (W) of electrolyte ions. The observed values reveal that a good thickness of Co(OH)_2_ covered on NiCo_2_O_4_ nanoparticles could enhance the electrochemical activities of the electrode. Figure S4a displays Nyquist plots and Figure S4b Cycling stability of Co(OH)_2_ electrode at 3 A·g^−1^ in the three-electrode electrochemical test.

**Figure 6:**
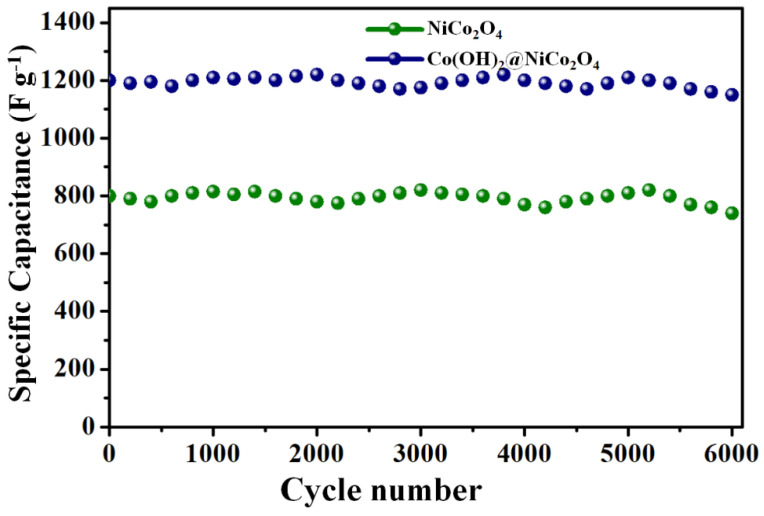
Electrochemical performances of cycling performance at the current density of 3 A·g^−1^ for composite Co(OH)_2_@NiCo_2_O_4_ nanosheet and pure NiCo_2_O_4_ electrode.

The long cycling stability is one of the significant parameters of SCs. To evaluate the stability of the composite Co(OH)_2_@NiCo_2_O_4_ nanosheet electrode and pure NiCo_2_O_4_ electrode, the samples were analyzed using GCD at 3 A·g^−1^. As illustrated in Figure 6, after 6000 cycles, the capacity retention remained at 92.83%, indicating superior reversible kinetics and cycling stabilities. The surface between the electrolyte and electrode would infiltrate through the ionic solution with restraining diffusions, and the material samples would improve the active sites via ‘ions’ diffusions with electrolytes and electrodes [48]. The specific capacitance of the as-prepared Co(OH)_2_@NiCo_2_O_4_ nanosheet electrode value is larger than that of the previously reported SCs, as shown in Table 1

## 4. Conclusions

In summary, the composite Co(OH)_2_@NiCo_2_O_4_ nanosheet samples on Ni foam were successfully prepared via easy hydrothermal preparation. The elemental compositions and higher crystallinities were validated using XPS and XRD analyzers. The morphological structure analysis of the Co(OH)_2_@NiCo_2_O_4_ nanosheet was carried out using SEM and TEM experiments. The NiCo_2_O_4_ nanoparticles components cohabited with the aggregations of Co(OH)_2_ particles in unique constructions. Owing to the porous constructions and the synergistic effects of NiCo_2_O_4_ and Co(OH)_2_, the as-designed composite Co(OH)_2_@NiCo_2_O_4_ nanosheet electrode achieved higher specific capacitance of 1308 F·g^−1^ at 0.5 A·g^−1^ and a notable cycle stability with 92.83% capacity retention over 6000 long-cycles. All the outcomes substantiate that composite Co(OH)_2_@NiCo_2_O_4_ nanosheet electrode is a promising candidate for wider applications prospects in high-performance SCs.

## Figures and Tables

**Table 1:** Comparison of the performances of recently reported NiCo_2_S_4_-based nanomaterials for supercapacitor applications in a three-electrode configuration.

Electrode	Fabrication Method	Electrolyte	Capacitance (Current Density)	Cycling Stability (No. of Cycles)	Ref.
NiCo_2_O_4_@CoxNi1-x (OH)_2_	electrochemical deposition	1 M KOH	5.71 F·cm^−1^ (5.5 mA·cm^−2^)	80% (3000 cycles)	[49]
carbon nanotube/NiCo_2_O_4_	electrochemical deposition	6 M KOH	694 F·g^−1^ (1 A·g^−1^)	91% (1500 cycles)	[50]
Ni(OH)_2_@NiCo_2_O_4_	electrochemical deposition	1 M KOH	5.71 F·cm^−1^ (2 mA·cm^−2^)	36% (1000)	[51]
graphene/NiCo_2_O_4_	electrochemical deposition	3 M KOH	1950 F·g^−1^ (7.5 A·g^−1^)	92.8% (10,000 cycles)	[52]
NiCo_2_O_4_@graphene nanoarchitectures	Hydrothermal	2 M KOH	778 F·g^−1^ (1 A·g^−1^)	90% (10,000 cycles)	[53]
NiCo_2_O_4_–RGO composite	Self-assembly	6 M KOH	835 F·g^−1^ (1 A·g^−1^)	80% (4000 cycles)	[54]
Co(OH)_2_@FeCo_2_O_4_	Hydrothermal	6 M KOH	1173.43 F·g^−1^ (1 A·g^−1)^	95.4% (5000)	[55]
sponge-like NiCo_2_O_4_/MnO_2_ ultrathin nanoflakes	electrochemical deposition	1 M KOH	935 F·g^−1^ (1 A·g^−1)^	103.1% (25,000)	[56]
**Co(OH)_2_@NiCo_2_O_4_ nanosheet**	**Hydrothermal**	**2 M KOH**	**1308 F·g^−1^ (0.5 A·g^−1^)**	**92.83% (6000)**	**This work**

## Data Availability

No new data were created or analyzed in this study. Data sharing is not applicable to this article.

## Supplementary Materials

The following supporting information can be downloaded at: https://www.mdpi.com/article/10.3390/nano13131981/s1, Figure S1. XPS full survey spectrum of Co(OH)2 and NiCo2O4 electrode materials. Figure S2 FTIR spectra of the as-prepared NiCo_2_O_4_ electrode (a), and Co(OH)_2_@NiCo_2_O_4_ nanosheets electrode (b). Figure S3. (a) CV curves and (b) CD plots of pure NiCo_2_O_4_ electrode at various scan rates and different current densities grown on Ni foam, and Figure S4. (a) Nyquist plots, and (b) Cycling stability of Co(OH)_2_ electrode material in three-electrode electrochemical test.
