# Supplementary material for: A Facile Two-Step Hydrothermal Synthesis of Co(OH)2@NiCo2O4 Nanosheet Nanocomposites for Supercapacitor Electrodes
Source: Nanomaterials (Basel). 2023 Jun 30;13(13):1981. doi: 10.3390/nano13131981 (PMC10343819; doi:10.3390/nano13131981)
Supplement: Supplementary file 1 [file nanomaterials-13-01981-s001.zip › nanomaterials-2398432-supplementary.pdf]

Supplementary Materials

A Facile Two-step Hydrothermal Synthesis of $Co(OH)_2@NiCo_2O_4$ nanosheet Nanocomposites for Supercapacitor Electrodes

Hammad Mueen Arbi [1,†], L. Vijayalakshmi [2,†], Yedluri Anil Kumar [3,4], Salem Alzahmi [3,4],* Chandu V. V. Muralee Gopi[5], Andrivo Rusydi[6], and Ihab M. Obaidat [1,4,*]

[1] Department of Physics, United Arab Emirates University, Al Ain 15551, United Arab Emirates; 201990023@uaeu.ac.ae (H.M.A.); yedluri.anil@gmail.com (Y.A.K.)

[2] Department of Automotive Engineering, Yeungnam University, Gyeongsan-si, Republic of Korea; lvl.phy@gmail.com

[3] Department of Chemical & Petroleum Engineering, United Arab Emirates University, Al Ain 15551, United Arab Emirates

[4] National Water and Energy Center, United Arab Emirates University, Al Ain 15551, United Arab Emirates

[5] Department of Electrical Engineering, University of Sharjah, Sharjah P.O. Box 27272, United Arab Emirates; naga5673@gmail.com

[6] Advanced Research Initiative for Correlated-Electron Systems (ARiCES), Department of Physics, National University of Singapore, Singapore 117551, Singapore, phyandri@nus.edu.sg

* Correspondence: s.alzahmi@uaeu.ac.ae (S.A); iobaidat@uaeu.ac.ae (I.M.O.)

† These authors contributed equally to this work.

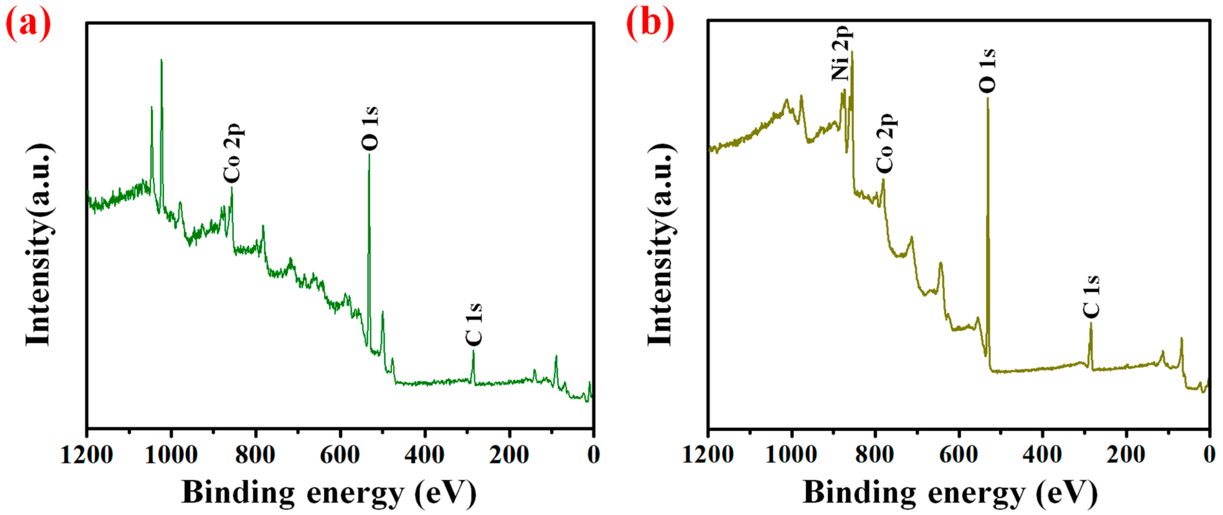

Figure S1. XPS full survey spectrums of (a) $Co(OH)_2$ and (b) $NiCo_2O_4$ electrode materials.

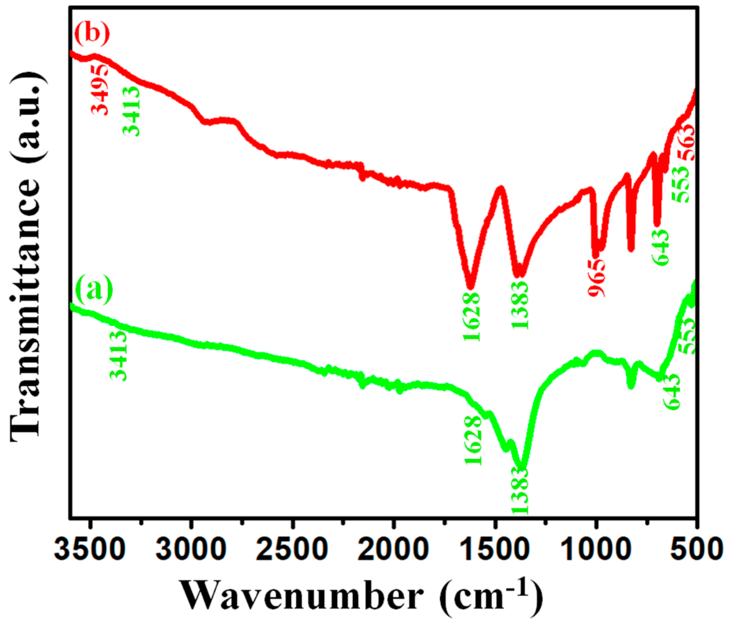

Figure S2. FTIR spectra of the as-prepared $NiCo_2O_4$ electrode (a) and $Co(OH)_2$@$NiCo_2O_4$ nanosheets electrode (b).

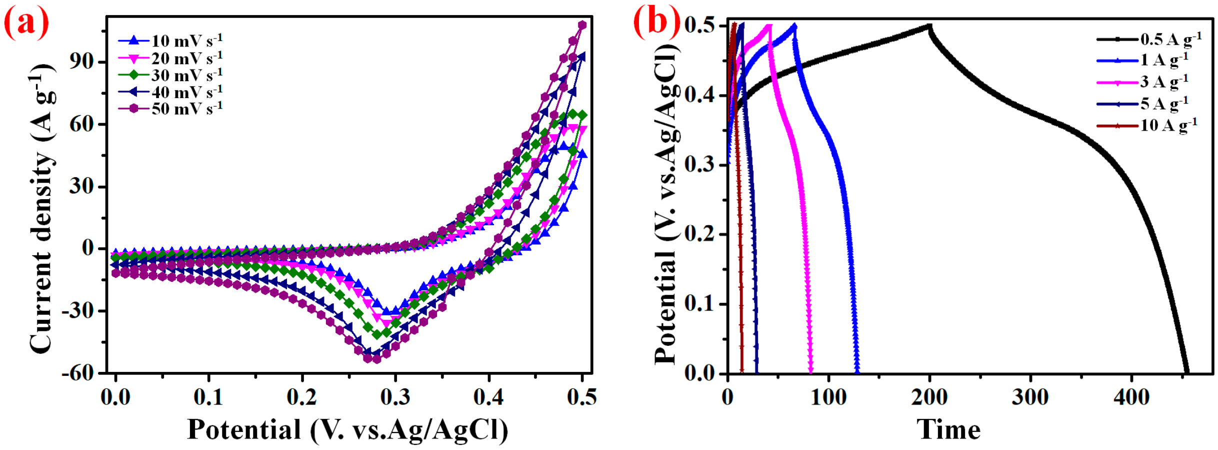

Figure S3. (a) CV curves and (b) CD plots of pure $NiCo_2O_4$ electrode at various scan rates and different current densities grown on Ni foam.

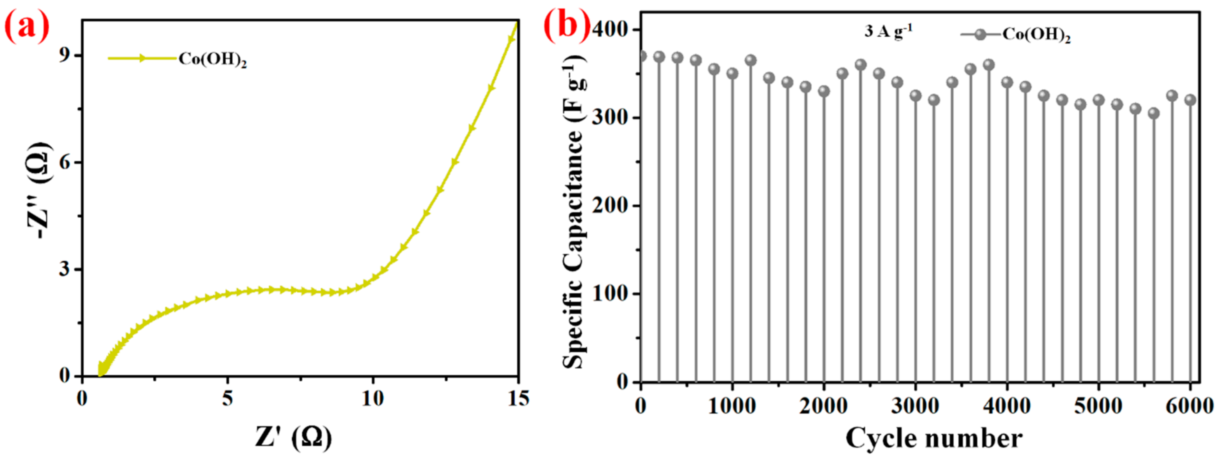

Figure S4. (a) Nyquist plots, and (b) Cycling stability of $Co(OH)_2$ electrode in three-electrode electrochemical test.
